# Phosphorylase Kinase β Represents a Novel Prognostic Biomarker and Inhibits Malignant Phenotypes of Liver Cancer Cell

**DOI:** 10.7150/ijbs.33278

**Published:** 2019-09-07

**Authors:** Wenjing Yang, Chunyan Zhang, Yihao Li, Anli Jin, Yunfan Sun, Xinrong Yang, Beili Wang, Wei Guo

**Affiliations:** 1 Department of Laboratory Medicine, Zhongshan Hospital, Fudan University, 136 Yi Xue Yuan Road, Shanghai 200032, P. R. China.; 2 Department of Liver Surgery, Liver Cancer Institute, Zhongshan Hospital, Fudan University; Key Laboratory of Carcinogenesis and Cancer Invasion, Ministry of Education, Shanghai 200032, P. R. China.

**Keywords:** *PHKB*, prognostic indicator, epithelial-mesenchymal transition, glycogen breakdown, signaling pathway

## Abstract

Glycogen phosphorylase kinase β-subunit (PHKB) is a regulatory subunit of phosphorylase kinase (PHK), involving in the activation of glycogen phosphorylase (GP) and the regulation of glycogen breakdown. Emerging evidence suggests that PHKB plays a role in tumor progression. However, the function of PHKB in HCC progression remains elusive. Here, our study revealed that the expression of PHKB significantly decreased in HCC tissues, and the low expression of PHKB could serve as an independent indicator for predicting poor prognosis in HCC. Functional experiments showed that PHKB knockdown significantly promoted cell proliferation both *in vitro* and *in vivo*, whereas PHKB overexpression resulted in opposing effects. Additionally, *in vitro* assays revealed that the over (or high) expression of PHKB greatly hindered HCC cell invasion and increased apoptosis rates. Also, we found that the over (or high) expression of PHKB effectively suppressed the epithelial-mesenchymal transition, which was further confirmed by our clinical data. Intriguingly, the biological function of PHKB in HCC was independent of glycogen metabolism. Mechanically, PHKB could inhibit AKT and STAT3 signaling pathway activation in HCC. Collectively, our data demonstrate that PHKB acts as a novel prognostic indicator for HCC, which exerts its suppression function via inactivating AKT and STAT3. Our data might provide novel insights into progression and facilitate the development of a new therapeutic strategy for HCC.

## Introduction

Hepatocellular carcinoma (HCC) is one of the most prevalent solid tumors worldwide, with over 750,000 deaths per year. Although treatments with chemotherapy, radiotherapy, surgical resection and biotherapeutics for HCC have achieved significant progress, the overall prognosis remains unfavorable due to the high incidence of recurrence or metastasis after treatment 1. Therefore, to improve the prognosis of HCC patients and understand the molecular pathogenesis of HCC, it is essential for researches to explore new biomarkers and understand their functional roles in HCC.

Glycogen metabolism has been regarded as a critical pathway involved in cancer metabolism reprogramming 2. Dysfunction and altered expression of the corresponding proteins and enzymes associated with glycogen conversion may be attributed to liver tumorigenesis. Glucose transporters (GLUTs), glycogen synthase kinase 3β (GSK-3β), and glycogen phosphorylase (GP) could undergo changes in structure and expression levels, regulating the entire glycogen metabolism pathway, which greatly contributed to liver tumor growth 3-5. Phosphorylase kinase (PHK) regulates glycogenolysis by activating GP, which catalyzes phosphorolytic degradation of glycogen. Glycogen phosphorylase kinase β-subunit (PHKB) is one of the regulatory subunits of PHK, which is necessary to activate the catalytic subunit γ of PHK and form PHK structure 6, 7. Masato *et.al* found that KIAA1199 can interact with PHKB to promote glycogen degradation and further sustain cancer cell survival 8. PHKB has also shown to be a novel prognostic biomarker in colorectal cancers and a promising anti-angiogenic therapeutic in the zebrafish model 9, 10. Thus, PHKB may be associated with glycogen metabolism and regulate cancer cell growth. However, the prognostic value and the biological function of PHKB in HCC remain elusive.

We report here that the protein expression of PHKB is decreased in HCC tissues when compared to the paired adjacent non-cancerous liver tissues. The low expression of PHKB is associated with poor prognosis in HCC patients. Modulations of PHKB expression in HCC cells exerted significant effects on proliferation, apoptosis, and motility capacities. Moreover, PHKB expression impressively suppressed epithelial-mesenchymal transition (EMT). Intriguingly, we found PHKB exerted its suppression functions in a glycogen phosphorylase independent manner. Further experiments identified STAT3 and AKT signaling as the key downstream pathway underlying PHKB in HCC cells.

## Methods and Materials

### Patients

Three independent cohorts of HCC patients were enrolled in present study from Zhongshan Hospital, Fudan University, i) 20 HCC patients received curative resection were recruited for PHKC expression evaluation in 2018. Fresh tumor tissues and their corresponding peritumoral liver tissues were collected. ii) Tissue microarrays (TMA) including 189 HCC tissues used in our study were collected from 2012 to 2013. iii) 13 HCC tissues were recruited from April 2019 to July 2019 to detect p-STAT3/p-AKT expression. HCC patients without radiotherapy and chemotherapy underwent surgical operation were recruited for present study. Hematoxylin and eosin (HE) staining was used to validate HCC diagnosis. Written informed consent was received from each patient enrolled in present study. Comprehensive clinicopathologic and follow-up information were collected. Overall survival (OS) was defined as the time between the date of surgical operation and death. Time-to-recurrence (TTR) was defined as the time interval between the date of surgery and the date when first evidence of recurrence was obtained. Follow-up ended in December 2017.

### Tissue Microarray analysis

Tissue microarrays (TMA) including 189 HCC tissues were established by a tissue microarrayer (Outdo Biotech, Shanghai, China). The primary antibody specific to PHKB (1:50, Abcam, Cambridge, MA) were applied for immunohistochemistry of TMA. Other primary antibodies, including E-cadherin (1:100, Abcam, Cambridge, MA) and N-cadherin (1:100, Abcam, Cambridge, MA), were also used. All slides were incubated with the secondary antibody (goat anti-mouse) next day. Immunohistochemical staining of the three genes was evaluated by two investigators, respectively. The staining of PHKB was mainly cytoplasmic in positive cases, whereas the expression of E-cadherin and N-cadherin was primarily detected on the cell membrane. Scores of all genes were depended on proportion score and intensity score. The proportions of the three genes staining were scored as follows: <10% scored 0, 10%-40% number of positive cells scored 1, 40%-70% scored 2 and >70% scored 3. The staining intensity was scored as follows: the negative scored 0, the weak scored 1, the intermediate scored 2, and the strong scored 3. When the cumulative intensity and percentage score exceeds 8 or more, the expression status was regarded as high.

### Cell culture, transfection and transduction

HCC cell lines, including HCCLM3, SMMC-7721, MHCC97H and Hep3B were purchased from the Chinese Academy of Sciences (Shanghai, China). Cells were cultured in Dulbecco's modified Eagle's medium (DMEM, Gibico BRL, Grand Island, NY) and added with 10% fetal bovine serum (Gibico BRL, Grand Island, NY) at 37°C. Short hairpin RNAs (shRNAs) were constructed via Shanghai GenePharma Co., Ltd (Shanghai, China). The sequences targeted PHKB: shPHKB#1: sense 5'-CCAGCUGACUUUGUAGAAUTT-3', antisense 5'-AUUCUACAAAGUCAGCUGGTT-3'; shPHKB#2: sense 5'-GGAUGUCAACAUUAGUGAATT-3', antisense 5'-UUCACUAAUGUUGACAUCCTT-3'; The negative controls (NC) shRNA: 5′-UUCUCCGAACGUGUCACGUtt-3′. shPHKB#1 and shPHKB#2 were transfected into HCCLM3 and SMMC-7721 cells using lipo2000 following manufactural instruction (Invitrogen, Carlsbad, CA). SMMC7721 cells with stable clones were selected with 600µg/mL puromycin (Sigma-Aldrich, St. Louis, USA) for 2 months approximately. The cDNA (NM_000293) sequence was synthesized by Genechem, and the Lenti-OE^TM^ vector was used to construct cells with PHKB overexpression. MHCC97H cells with stable PHKB expression were established.

### RT-PCR

The cancer tissues and non-cancerous tissues used in our study were from 20 patients with HCC. RNA Isolation Kits (Qiagen, Inc, Valencia, CA) were used to extract RNA from tissues and cells. Reverse transcription kits (Qiagen, Inc, Valencia, CA) were applied to convert isolated RNA into cDNA. The mRNA expression levels of PHKB were detected using SYBR Mix (Takara, Japan) and determined by Roche Real-time PCR Detection System (Roche Diagnostics). Primer details were available in supplementary materials (Supplementary Table 1).

### Western blot

The experiment was carried out as the previous study mentioned 11. Primary antibodies specific to PHKB (1:2000, Abcam, Cambridge, MA), PYGL (1:300, Abcam, Cambridge, MA), GYS1, p-AKT, AKT, p-Stat3, Stat3, p-Smad2/3, Smad2/3, p-ERK1/2, ERK1/2 (1:1000, CST, Boston, MA) were used.

### Assays of cell proliferation, invasion, and apoptosis in vitro

Cell Counting Kit-8 (Beyotime, Shanghai, China) was used to determine cell proliferation and transwell chambers (BD, PharMingen, San Jose, CA) with matrigel (Corning Inc, Corning, NY) were performed to evaluate invasion capacity. Cells were treated with Annexin V-FITC Apoptosis Detection Kit (BD, PharMingen, San Jose, CA) and then the rate of cell apoptosis was detected by flow cytometry (BD, Biosciences). The aforementioned experiments were operated according to the kit instructions and repeated for at least three times.

### Glycogen Quantification

DAB (1,4-dideoxy-1,4-imino-D-arabinitol hydrochloride) chemicals were purchased from Sigma (St. Louis, MO, US). Intercellular glycogen expression was examined using the Glycogen Assay Kit (BioVision, Milpitas, CA), followed as the manufacturer's instructions. In brief, cells were collected in a tube on ice and were added 200ul of dH_2_O to homogenize. After boiled at 95°C for 5 min, the tubes were centrifuged at 13,000 rpm for 5 min. The glycogen level of the supernatant collection was measured, which was normalized by protein content.

### Periodic Acid Schiff (PAS) Staining

Liver cancer cells were fixed using paraformaldehyde (4%) for 15min and then incubated using Periodic acid (1%) for 5min. After rinsing gently with water, the cells were treated with Schiff's regent for 20min. Finally, the cells were washed with running water for 5min and counterstained by Hematoxylin solution.

### Glucose Uptake Assay

The 24-well plate was used to culture liver cancer cells. After culturing overnight, the media was removed and then replaced with glucose-free DMEM containing 100µM 2-NBDG. After incubated in 37°C and 5% CO2 for one hour, the cells were washed twice with PBS. The 2-NBDG of glucose uptake was observed by microscope and flow cytometry.

### In Vivo Assay

Male nude mice (4-week-old) used in our study were from the department of Experimental Animals of the Chinese Academy of Sciences (Shanghai, China), which were divided into four groups with random allocation (n=6 per group). SMMC-7721 cells (5x10^6^) with PHKB knockdown were injected subcutaneously into the right side of the first group of nude mice. While parental SMMC-7721 cells as the second group were controls. MHCC97H cells (5x10^6^) with PHKB overexpression and PHKB-NC were subcutaneously injected into mice of the third and fourth groups, respectively. Tumor volume was measured every 4 days. After inoculation for 36 days, the tumors were detached and weighed from the mice. All animal procedures were conducted under the guidelines approved by the Institutional Animal Care and Use Committee (IACUC) at Zhongshan Hospital, Fudan University.

### Statistics

We used SPSS/Graph pad for statistical analysis, and P<0.05 was considered statistically significant. The relationships between clinicopathological parameters and PHKB were compared by X^2^ test and Fisher's exact test. The Kaplan-Meier method and the log-rank test were used to assess the overall survival and tumor recurrence rates of HCC patients. Prognostic parameters were assessed using univariate and multivariate analysis via establishing cox proportional hazards regression models, Spearmen's rank correlation analysis was used to evaluate the relationship between PHKB and EMT related markers.

## Results

### Decreased expression of PHKB in HCC tissues predicts a poor prognosis

To evaluate the clinical significance of PHKB expression in HCC, mRNA expression levels were examined in 20 human HCC tissues and paired noncancerous tissues via real-time PCR.

**Figure 1:**
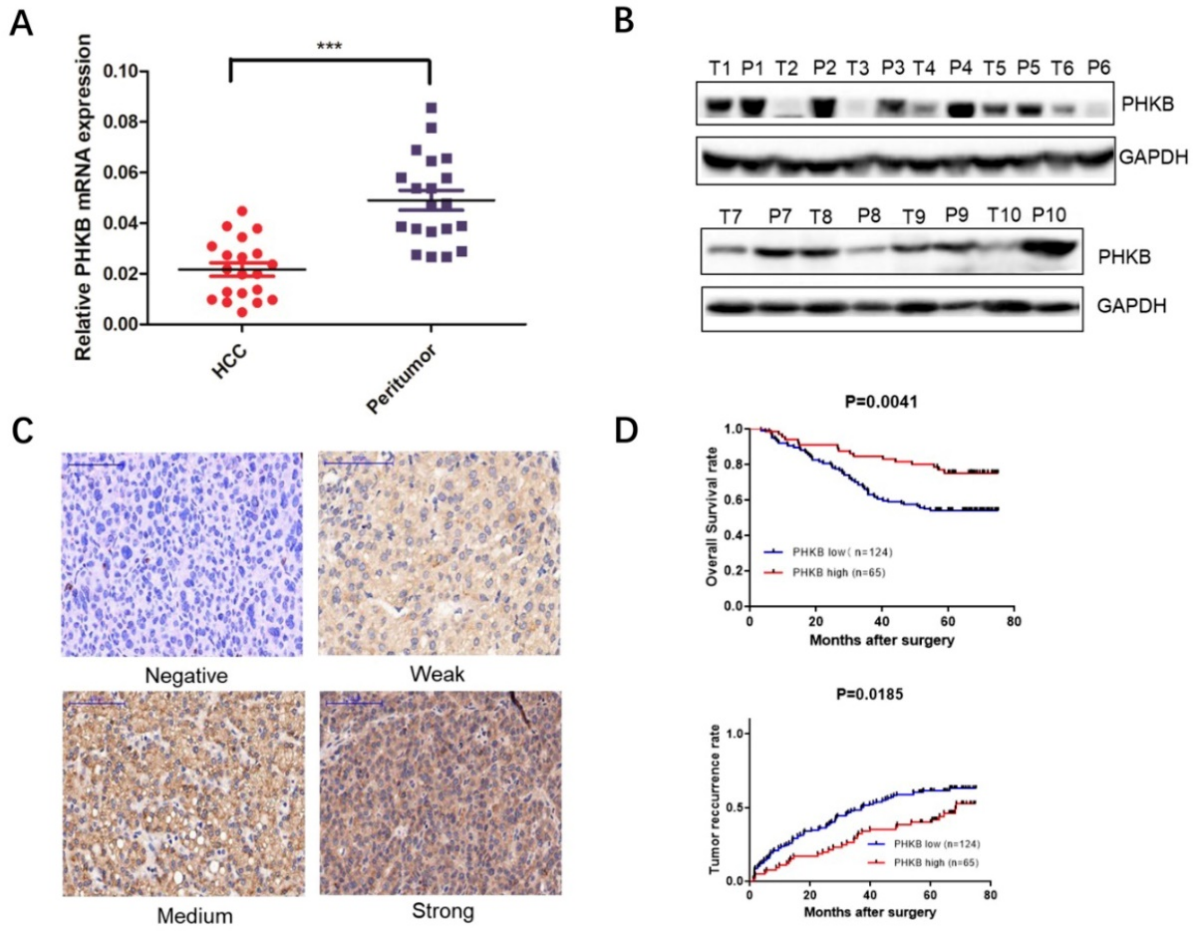
** Decreased PHKB expression was associated with poor outcomes of human HCC.** (A) The expression of PHKB mRNA was detected in 20 HCC tissues and the paired non-cancerous tissues. (B) The protein expression of PHKB was determined in 10 pairs of HCC tumor tissues (T) and corresponding peritumor tissues (P). (C) Representative images of PHKB immunohistochemistry in TMA slides were shown, including staining negative, weak, medium and strong. (D) Kaplan-Meier analysis of the overall survival and time to recurrence evaluated with a tissue microarray containing 189 HCC patients. Scale bar: 100μm.

Results showed that PHKB was down-regulated in most HCC tissues (Fig. 1A). Western blot (WB) assays further confirmed our findings from RT-PCR that PHKB protein expression levels decreased in most of HCC tissues when compared to paired non-cancerous liver tissues (Fig. 1B). Furthermore, TMA was performed to determine PHKB protein levels in 189 HCC patients, of which 124 (65.6%) patients were classified as low PHKB expression, and the remaining 65 (34.4%) patients were considered as high PHKB expression (Fig. 1C). Kaplan-Meier analysis showed that low PHKB group had significantly shorter overall survival (P=0.0041) and time-to-recurrence (P=0.0185) than those of high PHKB group (Fig. 1D). Correlations between PHKB expression and clinicopathological characteristics were also evaluated. As listed in Table 1, low PHKB expression was closely associated with tumor number (P=0.015), microvascular invasion (P=0.030), and BCLC stage (P=0.019). Univariate analysis showed that macrovascular invasion (RR=2.19, 95%CI=1.13-4.28, P=0.021), microvascular invasion (RR=1.91, 95%CI=1.21-3.04, P=0.006), BCLC stage (RR=1.72, 95%CI=1.01-2.94, P=0.045) and PHKB (RR=2.212, 95%CI=1.269-3.846, P=0.005) were associated with recurrence and overall survival of HCC patients (Table 2). Importantly, multivariate analysis confirmed that lower PHKB expression was an independent prognostic predictor for shorter OS (Table 3; RR=2.083, 95%CI=1.449-4.808, P=0.002) and TTR (Table 3; RR=1.934, 95%CI=1.239-3.012, P=0.004) in HCC patients.

### PHKB inhibited cell proliferation and induced cell apoptosis

**Figure 2:**
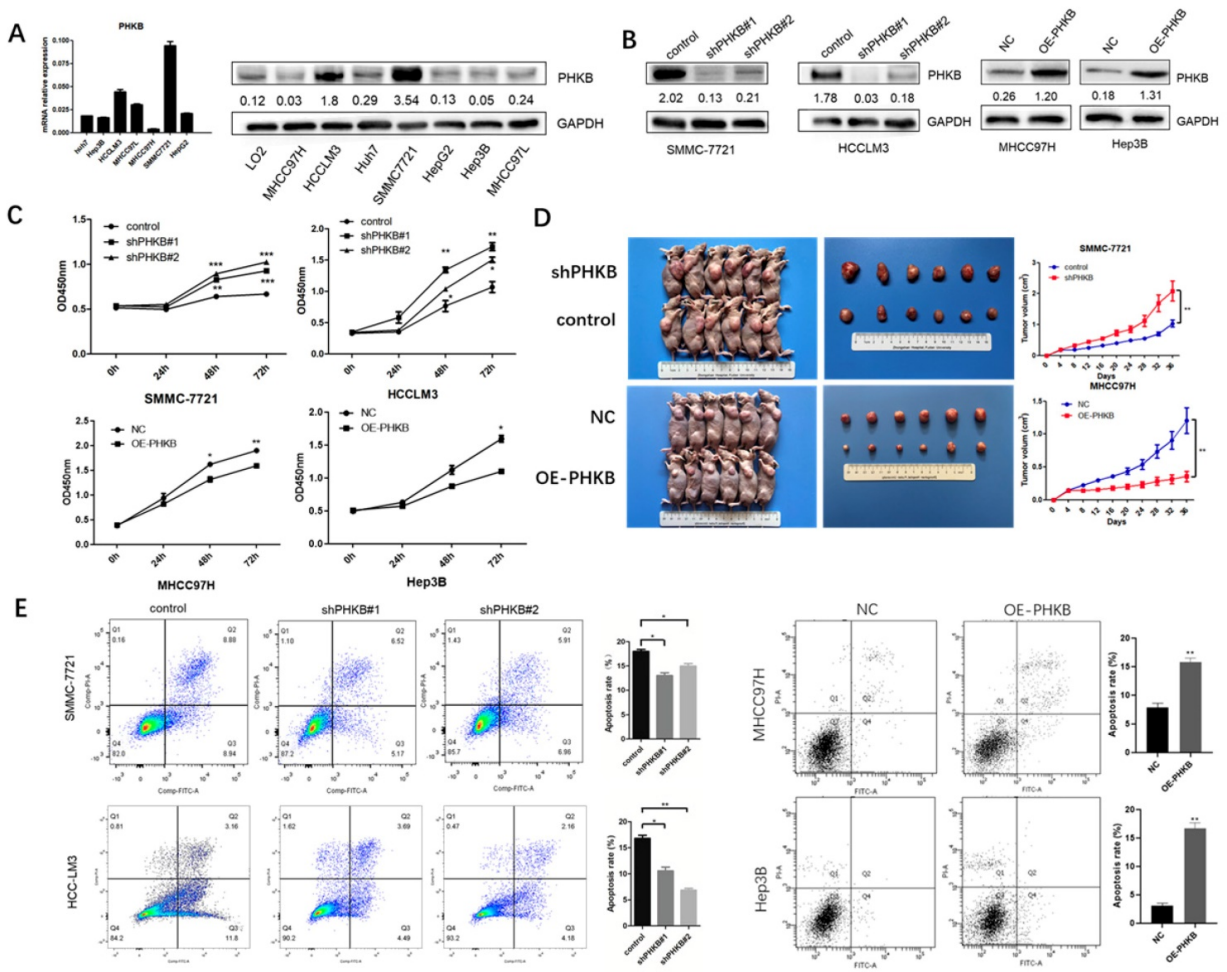
** PHKB inhibited the liver cancer cell proliferation* in vitro* and* in vivo*.** (A) The expression level of PHKB was analyzed in 7 HCC cell lines. (B) PHKB knockdown and overexpression efficiencies in indicated HCC cell lines were evaluated by WB assays. (C) *In vitro* effects of PHKB on HCC cell proliferation were detected by CCK-8 kit. (D)* In vivo* effects of PHKB on HCC proliferation*.* (E) Effects of PHKB expression on HCC cell apoptosis were assayed by flow cytometry. Data were shown as means +SD from three independent experiments. *P<0.05, **P<0.01, and ***P<0.001.

Based on the relationship between PHKB expression and tumor clinicopathological characteristics, we found that the decrease in PHKB expression was closely correlated with aggressive features. Therefore, we investigated whether PHKB could suppress HCC progression. The mRNA and protein expression of PHKB in HCC cell lines were determined (Fig. 2A). High expression of PHKB was down-regulated in HCCLM3 cells and SMMC-7721 cells whose endogenous PHKB expressions were high. While PHKB overexpression was performed in MHCC97H cells and Hep3B cells whose endogenous PHKB expressions were relatively low (Fig. 2B). To determine the role of PHKB in tumor proliferation, stable PHKB expression modulated cell lines were generated and their proliferation ability was analyzed *in vitro and in vivo. In vitro* experiment results revealed that down-regulation of PHKB promoted the growth of HCCLM3 cells and SMMC-7721 cells, whereas overexpression of PHKB inhibited the growth of MHCC97H cells and Hep3B cells (Fig. 2C). Consistently*,* mice injected with cells overexpressing PHKB showed a significant decrease in both tumor volumes and weights, while cells with down-regulated PHKB exhibited an increase in tumor volumes and weights (Fig. 2D). In addition, apoptosis was significantly restricted due to PHKB downregulation, whereas overexpression of PHKB resulted in opposing effects (Fig. 2E). Collectively, our data demonstrated that PHKB significantly hindered proliferation and promoted apoptosis in HCC cells.

### PHKB expression was associated with the EMT phenotype in HCC

**Figure 3:**
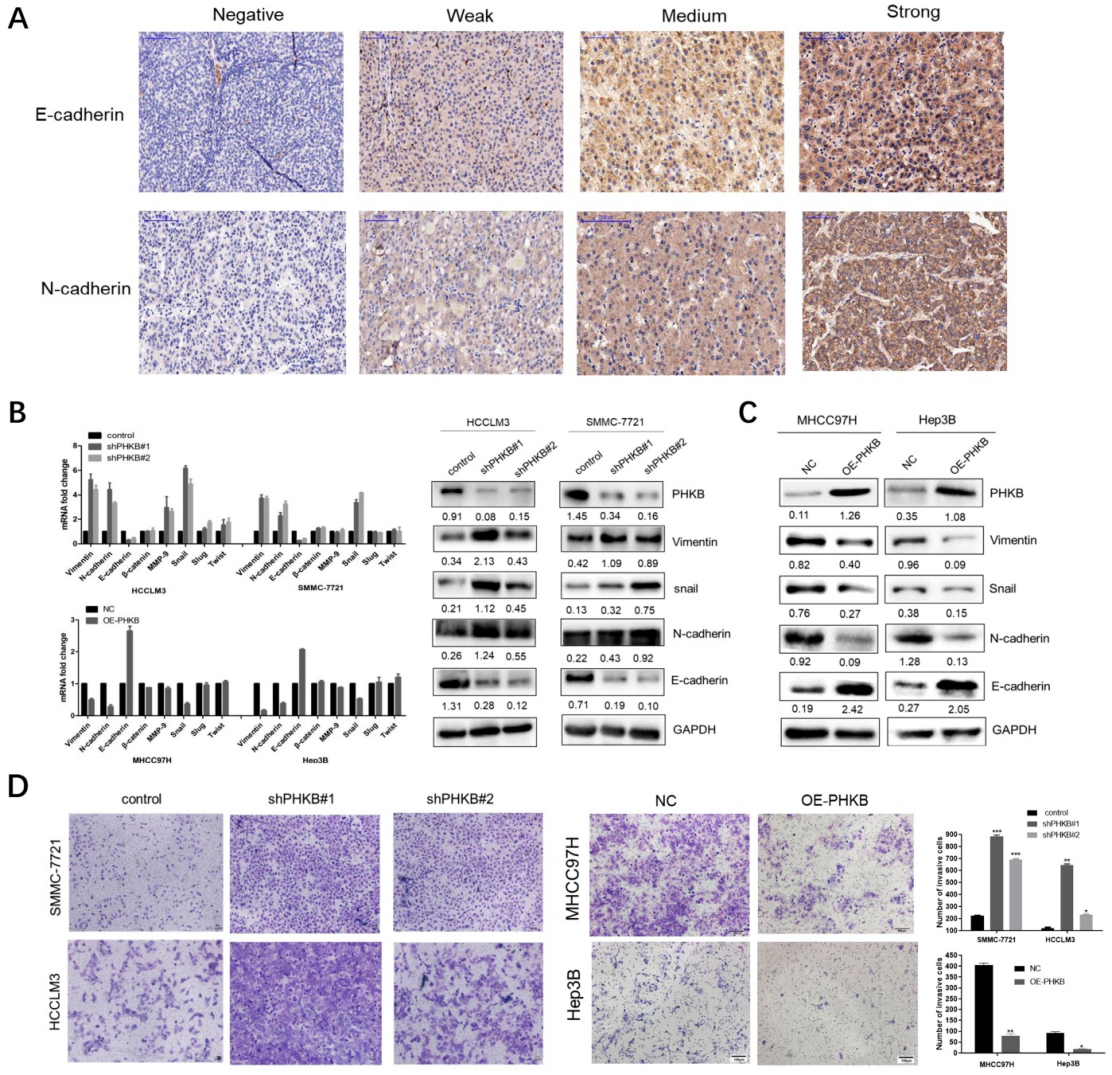
** PHKB expression suppressed EMT-like phenotypes.** (A) Representative images of E-cadherin and N-cadherin expression status in TMA slides were shown, including staining negative, weak, medium and strong. (B) mRNA and protein expression of EMT-related markers were detected in PHKB knockdown cells. (C) mRNA and protein expression of EMT-related markers were detected in PHKB overexpression cells. (D) Transwell assays showed the effects of PHKB expression on cell invasive capacities. Data were shown as means +SD from three independent experiments. *P<0.05, **P<0.01, and ***P<0.001. The value of the bar is 100μm.

Epithelial-mesenchymal transition is considered as a key process, which renders HCC cells with invasiveness potentials. Given that PHKB expression was associated with invasiveness phenotype in HCC, we further investigated the correlation between PHKB expression and EMT status by IHC staining in TMA. Typical staining images were shown in Fig. 3A. Spearman rank correlation analysis demonstrated that PHKB was positively associated with E-cadherin expression (r=0.179, p=0.013), while negatively correlated with N-cadherin expression (r=-0.173, P=0.017) (Table 4). In addition, qPCR and WB were performed to evaluate the alteration of EMT-related markers, including epithelial cell marker (E-cadherin), mesenchymal cell markers (N-cadherin, vimentin, and β-catenin), extracellular matrix protein (MMP9), and EMT-related transcription factors (slug, snail, and twist). Results showed that mRNA expressions of the mesenchymal-related markers (N-cadherin, vimentin, MMP9, slug) were significantly increased, but epithelial-related marker (E-cadherin) decreased in cells with PHKB knockdown. Four markers (N-cadherin, vimentin, E-cadherin, and snail) which showed significant mRNA alterations also had similar expression patterns at the protein level (Fig. 3B). Contrarily, cells with PHKB overexpression resulted in opposing effects according to qPCR and WB assays (Fig. 3C). To reinforce the molecular changes due to PHKB modulations, we further evaluated the effect of PHKB on cell invasive ability *in vitro* via Transwell assays. As we expected, PHKB knockdown resulted in significant enhancement of cell invasiveness potentials, while PHKB overexpression greatly reduced the number of invading cells (Fig. 3D). Therefore, our data revealed that PHKB might inhibit HCC invasion via suppressing EMT.

### The functions of PHKB were independent of glycogenolysis

**Figure 4:**
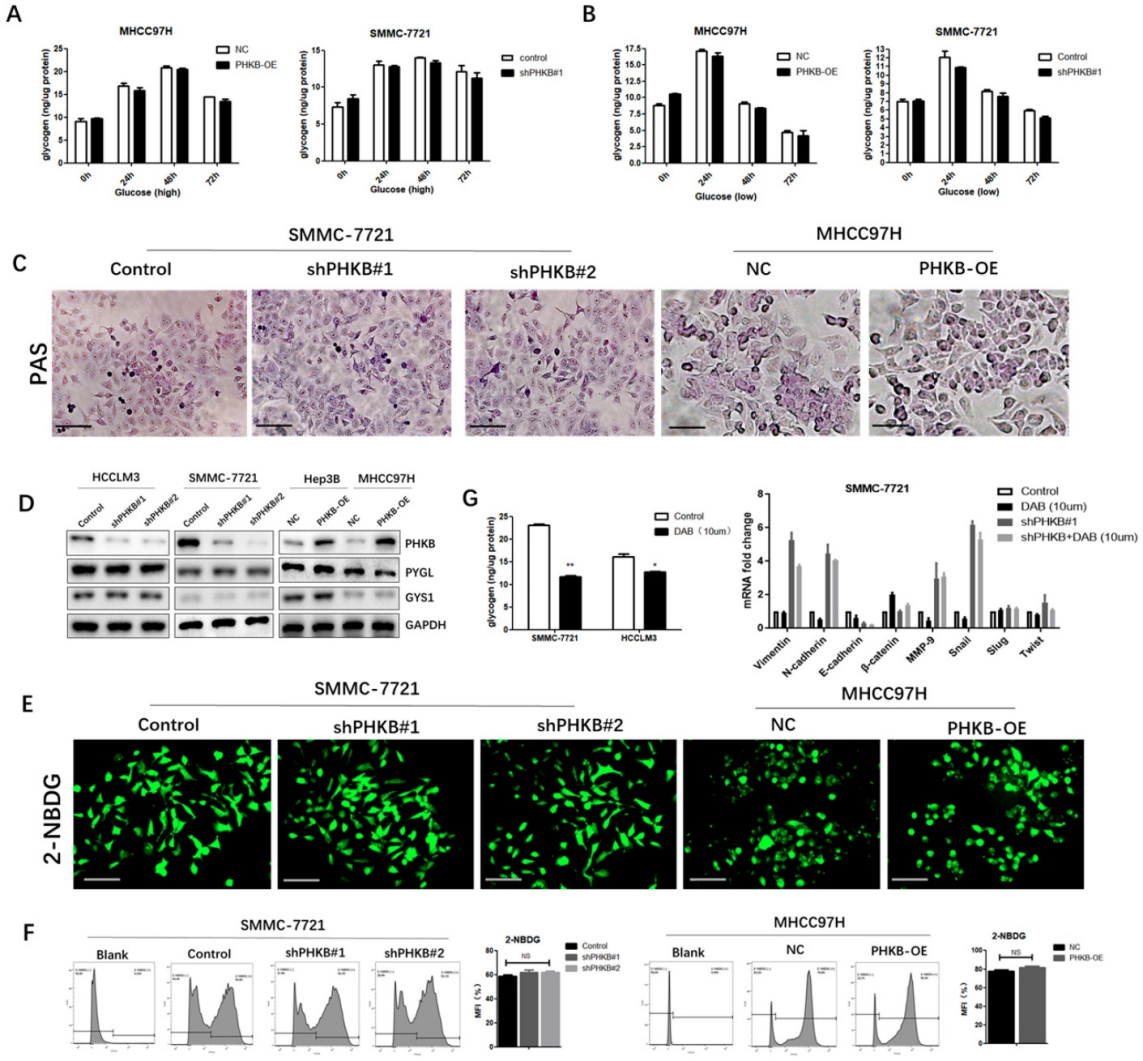
** PHKB expression may be independent of glycogen metabolism.** (A) Glycogen levels were determined in PHKB-OE MHCC97H cells and shPHKB SMMC-7721 cells cultured in high glucose conditions. (B) Glycogen levels were measured in PHKB-OE MHCC97H cells and shPHKB SMMC-7721 cells cultured in low glucose conditions. (C) The intracellular glycogen expression was detected by PAS staining. (D) PYGL and GYS1 protein expression were detected in PHKB knockdown cells as well as PHKB overexpressed HCC cells. (E) Glycose uptake potentials of indicated cells were evaluated with 2-NBDG. (F) Glycose uptake potentials of indicated cells were determined by flow cytometry. (G) Intracellular glycogen levels were determined after DAB treatment (left), and mRNA expression levels of EMT-related markers were detected by PCR assays after DAB (10uM) treatment in SMMC-7721 cells (right). Data were shown as means +SD from three independent experiments. *P<0.05, **P<0.01, and ***P<0.001. Scale bar: 200μm.

PHKB is one of the regulatory subunits of PHK complex, which serves as a catalytic enzyme of GP and promotes glycogen breakdown. Therefore, whether PHKB suppressed EMT via regulating glycogenolysis was further investigated. First, we measured the difference of intercellular glycogen content between high and low glucose culture condition. We observed distinct intracellular glycogen patterns that glycogen contents escalating to reach the highest peak at 48 hours, then decreased rapidly in high glucose condition (Fig. 4A), whereas glycogen levels peaked at 24h under low glucose condition, and then descended slowly (Fig. 4B). However, we found PHKB modulation exerted no significant effects on these intracellular glycogen dynamic changes, suggesting that expression of PHKB may not influence glycogen content (Fig. 4A and B). Besides, intracellular glycogen contents were also detected by Periodic Acid-Schiff staining. Consistent with previous results, PHKB modulations failed to alter intracellular glycogen contents (Fig. 4C). The relationship between PHKB expression and PYGL (glycogen phosphorylase) and GYS1 (glycogen synthase) expression were also evaluated by western blotting. In spite of PHKB expression modulations, PYGL and GYS1 protein levels remained unchanged (Fig. 4D). Furthermore, 2-NBDG staining was performed to evaluate the effects of PHKB on glucose uptake. No significant differences of fluorescence were observed, either in PHKB Knockdown or overexpression cells (Fig. 4E). These findings were further substantiated by flow cytometry assays (Fig. 4F). Collectively, the above results indicated that PHKB exerted no significant regulatory effect on glycogenesis in HCC cells. To further confirm PHKB regulated EMT in a glycogenolysis-independent manner, HCC cells were cultured in media supplemented with 10uM DAB, an inhibitor of glycogen synthesis in intact hepatocytes 12. Since the maximal level of glycogen breakdown occurred at 48h, liver cancer cells were cultivated for 48 hours. As shown in Fig. 4G, DAB treatment greatly hindered glycogenesis in HCC cells, evidenced by reduced intracellular glycogen content. Moreover, qPCR results showed that HCC cells exhibited no significant alterations after DAB treatment. More importantly, DAB treatment also failed to reverse the mesenchymal phenotype caused by PHKB knockdown (Fig. 4G).

### PHKB involved in HCC progression via STAT3 and AKT signaling

**Figure 5:**
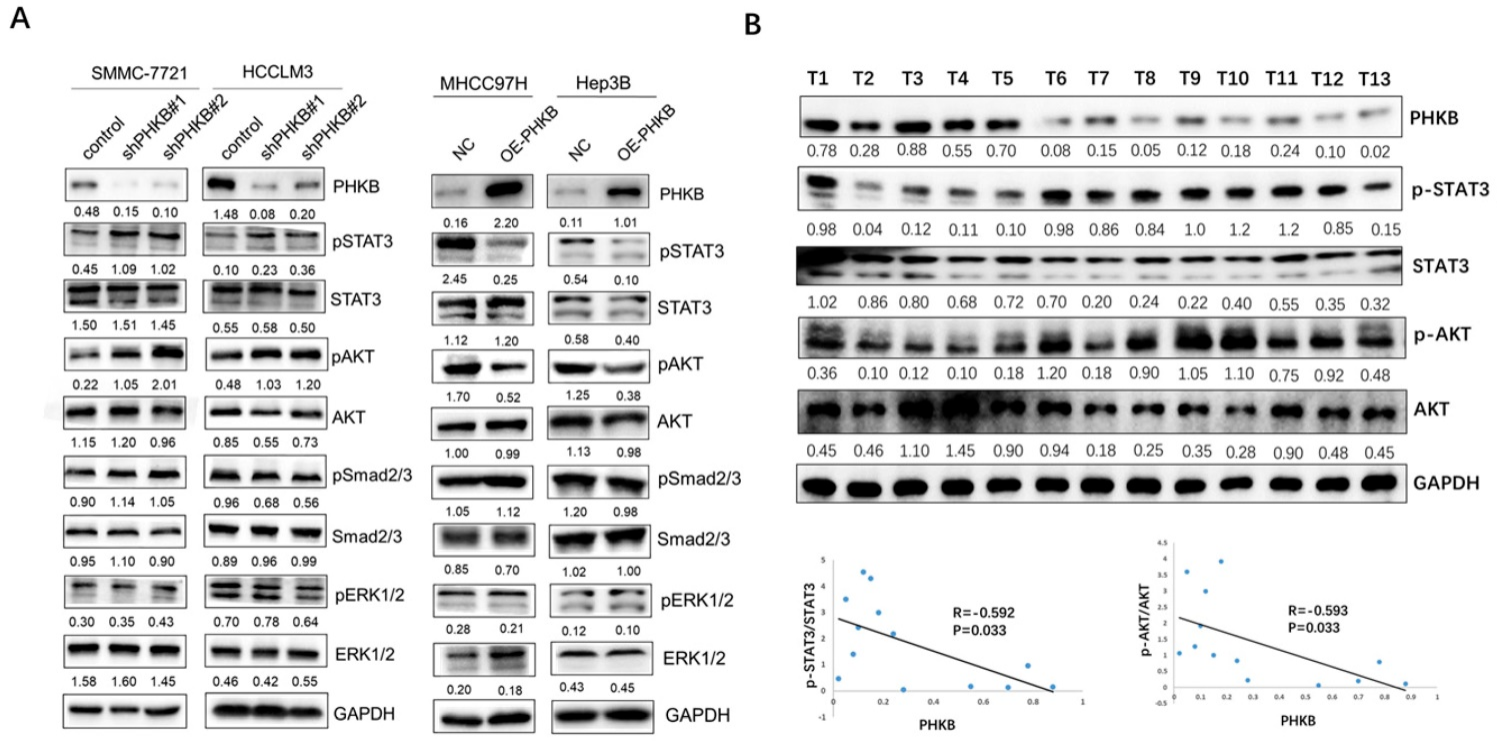
** PHKB expression was associated with AKT and STAT3 signaling in HCC cell lines.** (A) The expression levels of p-AKT, p-ERK1/2, p-STAT3 and p-Smad2/3 in PHKB knockdown cells as well as PHKB overexpressed cells were detected by western blotting. (B) The correlations between p-AKT/p-STAT3 expression and PHKB expression in HCC tissues.

Aberrant activation of AKT, ERK, STAT3, and Smad2/3 was reported to be closely associated with hepatocarcinogenesis and tumor progression 13-16. Here, we investigated the impacts of PHKB on these signaling pathways to identify the key downstream pathway underlying PHKB. As shown in Figure 5A, along with PHKB knockdown, phosphorylation of STAT3 and AKT increased in HCCLM3 cells and SMMC-7721 cells. However, overexpression of PHKB in Hep3B and MHCC97H cells resulted in decreased expression of phosphorylated STAT3 and AKT. The activity of ERK1/2 and Smad2/3 signaling was not affected by PHKB expression. Next, correlations between p-STAT3/p-AKT expression and PHKB were further evaluated in HCC tissue samples via WB assays (n=13). Results revealed that expression of PHKB was inversely correlated with both p-STAT3 and p-AKT levels (Fig. 5B). These results indicated that PHKB might suppress EMT and invasion of HCC cells via inactivating AKT and STAT3 signaling pathway.

## Discussion

Recently, numerous studies have reported that glycogen metabolism is reprogrammed in various cancers and plays a critical role in the pathophysiology of cancer cells 2. This process involves many enzymes and molecules that regulate glycogen synthesis and breakdown. Here, we identify the expression of PHKB, a molecule that is indirectly involved in the activation of glycogen phosphorylase, decreased in liver cancer cells. Low PHKB expression could serve as an independent indicator for predicting poor prognosis in HCC and enhanced the proliferation, invasion, and metastasis of liver cancer cells.

PHK was a hexadecameric complex consisting of α, β, γ, and δ subunits. Among them, α, β, and δ were regulatory subunits, and γ was a catalytic subunit, which was responsible for the enzymatic activation of PHK. Research suggested that beta subunit might be more vital for the biological function of HCC, compared with other subunits. Phosphorylation of PHKB has been found to activate PHKG, thereby resulting in the activation of PHK complexes and further stimulating GP function 8. Besides, mutations in PHKB in the liver are associated with glycogen storage diseases 17. Moreover, high expression levels of PHKB promoted cancer cell survival and showed a poor overall survival in patients with colorectal cancer, which was indirectly related to glycogen metabolism 8-9. However, there are few studies on phosphorylase kinase regulatory subunit alpha (PHKA), and there is no evidence showed that PHKA is involved in glycogen metabolism. The phosphorylase kinase regulatory subunit (PHKD), also known as calmodulin 3 (CALM), mainly binds Ca2+ and functions as an enzymatic cofactor 18. CALM was closely related to heart disease, rather than glycogen metabolism diseases 19.

Previous studies indicated that PHKB promoted biological functions such as cell proliferation by increasing glycogen breakdown in cancer cells 8. Therefore, the relationship between PHKB expression and glycogenolysis in HCC was explored. Glycogenolysis is essential ways in glycogen metabolism. Pancreatic tumor cells have shown to induce apoptosis by impairing glycogen degradation 20. GP is a key enzyme in glycogenolysis to maintain cell survival. The GP contains three isoforms, including muscle pattern (PYGM), liver pattern (PYGL) and the brain pattern (PYGB). Favaro and his colleagues found that the depletion of PYGL would trigger glycogen accumulation, induce premature senescence of cancer cells and strongly inhibit tumor growth 21. Changes in glycogen levels are another important characteristic of glycogenolysis. The glycogen content in cancer cells may undergo dramatic temporal changes. When exposed to hypoxic conditions, cancer cells would increase glycogen stores at first and provide energy production in the absence of nutrients 21-23. Therefore, we analyzed the correlation between PHKB expression and PYGL and glycogen levels to assess whether PHKB expression affected glycogenolysis. Our results showed that the alteration of PHKB expression had no influence on PYGL and glycogen expression, although glycogen contents decreased during nutrient deprivation. These observations indicated that PHKB expression was independent of the glycogenolytic pathway.

Our study for the first time showed that PHKB suppressed the growth of liver cancer cell. Although this observation was inconsistent with the function of PHKB in colorectal cancers, data from clinical patients indicated that PHKB was a potential tumor suppressor in HCC. The different function of PHKB between colorectal cancer and liver cancer may attributed to tumor heterogeneity, so even the same molecule may play different biological functions. In addition, the specific mechanism by which PHKB involves in colorectal cancer and liver cancer is diverse. In colorectal cancer, the authors believe that PHKB may promote glycogen degradation, thereby maintaining tumor cell survival 8. However, in liver cancer, we found that PHKB may be independent of glycogen metabolism, but be related to p-AKT and p-STAT3 signaling pathway. Furthermore, numerous studies have shown that the same gene has opposite biological functions in different research conditions and models 24. The tumor microenvironment associated with glycogen metabolism requires to be considered in our studies, such as hypoxic condition and acidic microenvironment. More importantly, it is unclear whether phosphorylated PHKB is involved in glycogenolysis. This is our subsequent project to clarify the function of PHKB.

To conclude, our paper was the first to identify the function of PHKB in HCC. We not only demonstrated the prognostic value of PHKB in patients with HCC, but we also investigated the function of PHKB* in vitro* and *in vivo*. More importantly, we found that PHKB expression did not influence glycogenolysis, but affected the activation of p-AKT and p-STAT3. Further investigation will be required to understand the role of phosphorylated PHKB and to fully describe the specific mechanisms of AKT and STAT3 signaling pathways.

## Supplementary Material

Supplementary table.

## Figures and Tables

**Table 1:** The correlations between PHKB expression and clinicopathological characteristics in HCC

Variables	N	PHKB expression		*p*-value
		Low (124)	high (65)	
Gender				
Male	151	98 (64.9)	53 (35.1)	0.683
Female	38	26 (68.4)	12 (31.6)	
Age (years)				
>50	110	75 (68.2)	35 (31.8)	0.379
≤50	79	49 (62.0)	30 (38.0)	
Child-Pugh score				
A	179	116 (64.8)	63 (35.2)	0.325
B	10	8 (80)	2 (20)	
Liver cirrhosis				
No	42	23 (54.8)	19 (45.2)	0.093
Yes	147	101 (30.6)	46 (69.4)	
ALT,U/L				
≤40	134	83 (33.6)	51 (66.4)	0.098
>40	55	41 (68.7)	14 (31.3)	
AST,U/L				
≤40	135	84 (62.2)	51 (37.8)	0.121
>40	54	40 (74.1)	14 (25.9)	
AFP, ng/ml				
≤400	140	92 (65.7)	48 (34.3)	0.959
>400	49	32 (65.3)	17 (34.7)	
Tumor number				
Single	169	106 (62.7)	63 (37.3)	**0.015**
Multiple	20	18 (90)	2 (10)	
Tumor size,cm				
≤5	119	77 (64.7)	42 (35.3)	0.733
>5	70	47 (67.1)	23 (32.9)	
Tumor margin				
Clear	178	116 (65.2)	62 (34.8)	0.609
Unclear	11	8 (72.7)	3 (27.3)	
Tumor encapsulation				
Complete	123	83 (67.5)	40 (32.5)	0.460
None	66	41 (62.1)	25 (37.9)	
Satellite lesion				
No	171	111 (64.9)	60 (35.1)	0.535
Yes	18	13 (72.2)	5 (27.8)	
Macro vascular invasion				
No	175	113 (64.6)	62 (35.4)	0.289
Yes	14	11 (78.6)	3 (21.4)	
Micro vascular invasion				
No	107	77 (72.0)	30 (38.0)	**0.030**
Yes	82	47 (56.8)	35 (43.2)	
Edmondson stage				
Ⅰ-Ⅱ	126	80 (63.5)	46 (36.5)	0.386
Ⅲ-Ⅳ	63	44 (69.8)	19 (30.2)	
BCLC stage				
0+A	158	98 (62.0)	60 (38.0)	**0.019**
B+C	31	26 (83.9)	5 (16.1)	

**Table 2:** Univariate cox proportional regression analysis of factors associated with recurrence and overall survival

		Recurrence			Overall survival	
Variables		HR (95% CI)	*P*		HR (95% CI)	*P*
Age (>50y versus ≤50y)		0.73 (0.50-1.07)	0.104		0.64 (0.41-1.02)	0.060
Sex (male versus female)		0.76 (0.48-1.21)	0.249		1.16 (0.69-1.95)	0.563
Liver cirrhosis (yes versus no)		1.52 (0.92-2.49)	0.099		1.11 (0.63-1.96)	0.712
ALT (>40U/L versus ≤40U/L)		1.57 (1.06-2.34)	**0.025**		1.8 (0.85-2.24)	0.190
AST (>40U/L versus ≤40U/L)		1.78 (1.20-2.64)	**0.004**		1.49 (0.92-2.41)	0.101
AFP (>400ng/ml versus ≤400ng/ml)		1.86 (1.25-2.78)	**0.002**		1.49 (0.91-2.43)	0.117
No. of tumors (multi versus single)		1.81 (1.06-3.08)	**0.030**		1.57 (0.84-3.02)	0.157
Tumor size (>5cm versus ≤5cm)		2.35 (1.60-3.43)	**<0.001**		1.59 (0.99-2.48)	0.056
Tumor encapsulation (none versus complete)		1.15 (0.78-1.69)	0.452		1.03 (0.64-1.67)	0.903
Satellite lesions (yes versus no)		1.67 (0.96-2.88)	0.069		0.78 (0.33-1.76)	0.528
Macro vascular invasion (yes versus no)		2.30 (1.28-4.12)	**0.005**		2.19 (1.13-4.28)	**0.021**
Micro vascular invasion (yes versus no)		2.08 (1.42-3.04)	**<0.001**		1.91 (1.21-3.04)	**0.006**
Edmondson stage (III-IV versus I-II)		1.78 (1.21-2.61)	**0.003**		1.50 (0.94-2.39)	0.087
BCLC stage (B+C versus 0+A)		1.98 (1.27-3.09)	**0.003**		1.72 (1.01-2.94)	**0.045**
ALBI grade (II versus I)		1.23 (0.80-1.89)	0.344		1.86 (1.12-3.09)	**0.017**
PHKB (Low versus high)		1.64 (1.082-2.494)	**0.020**		2.212 (1.269-3.846)	**0.005**

Abbreviations: ALT, alanine aminotransferase; AST, aspartate transaminase; AFP, α-fetoprotein; BCLC, Barcelona Clinic Liver Cancer; HR, hazard ratio; N.A, not applicable.

**Table 3:** Multivariate cox proportional regression analysis of factors associated with recurrence and overall survival

	Recurrence			Overall survival	
Variables	HR (95% CI)	*P*		HR (95% CI)	*P*
ALT (>40U/L versus ≤40U/L)	1.353 (0.857-2.134)	0.194		1.326 (0.754-2.331)	0.327
AST (>40U/L versus ≤40U/L)	1.332 (0.840-2.114)	0.223		1.149 (0.650-2.031)	0.634
AFP (>400ng/ml versus ≤400ng/ml)	1.934 (1.267-2.954)	**0.002**		1.542 (0.915-2.598)	0.104
No. of tumors (multi versus single)	4.458 (1.198-16.592)	**0.026**		5.604 (1.362-23.052)	**0.017**
Tumor size (>5cm versus ≤5cm)	1.608 (1.022-2.530)	** 0.040**		1.092 (0.627-1.899)	0.757
Macro vascular invasion (yes versus no)	5.324 (1.348-21.033)	**0.017**		7.879 (1.804-34.418)	**0.006**
Micro vascular invasion (yes versus no)	1.306 (0.811-2.105)	0.273		1.506 (0.846-2.682)	0.164
Edmondson stage (III-IV versus I-II)	1.526 (1.009-2.307)	**0.045**		1.194 (0.721-1.978)	0.491
BCLC stage (B+C versus 0+A)	0.267 (0.060-1.182)	0.082		0.200 (0.038-1.040)	**0.056**
ALBI grade (II versus I)	0.659 (0.398-1.090)	0.104		0.480 (0.271-0.850)	**0.012**
PHKB (Low versus high)	1.934 (1.239-3.021)	**0.004**		2.083 (1.449-4.808)	**0.002**

Abbreviations: ALT, alanine aminotransferase; AST, aspartate transaminase; AFP, α-fetoprotein; BCLC, Barcelona Clinic Liver Cancer; HR, hazard ratio; N.A, not applicable.

**Table 4:** The relationships between PHKB expression and EMT-markers in HCC.

Markers	N	PHKB expression		r	*p*-value
		High (%)	Low (%)		
E-cadherin	189	65	124		
High	90	39	51	0.179	**0.013**
Low	99	26	73		
N-cadherin					
High	101	27	74	-0.173	**0.017**
Low	88	38	50		
