# Supplementary material for: Phosphorylase Kinase β Represents a Novel Prognostic Biomarker and Inhibits Malignant Phenotypes of Liver Cancer Cell
Source: Int J Biol Sci. 2019 Sep 7;15(12):2596–606. doi: 10.7150/ijbs.33278 (PMC6854366; doi:10.7150/ijbs.33278)
Supplement: Supplementary file 1 — Supplementary table. [file ijbsv15p2596s1.pdf]

Supplementary Table 1. The primer sequences were used in study.

Gene		Sequences
PHKB	Forward	5′-CCCAGAGAGTTTCATTATCAGG-3′
	Reverse	5′-GTGAGCTGGAAGGTCTTGGA-3′
GAPDH	Forward	5′-GTCATCCAACGGGAATGCA-3′
	Reverse	5′-TGATCGGTTACCGTGATCAAAA-3′
E-cadherin	Forward	5′-TACGCCTGGGACTCCACCTA-3′
	Reverse	5′-CCAGAAACGGAGGCCTGAT-3′
N-cadherin	Forward	5’ATCCTACTGGACGGTTCG3’
	Reverse	5’TTGGCTAATGGCACTTGA3’
Vimentin	Forward	5’GAACGCCAGATGCGTGAAATG3’
	Reverse	5’CCAGAGGGAGTGAATCCAGATTA3’
β-catenin	Forward	5'-CAGAAGCTATTGAAGCTGAGG-3'
	Reverse	5’-TTCCATCATGGGGTCCATAC-3'
MMP-9	Forward	5′-GGGACGCAGACATCGTCATC-3′
	Reverse	5′-TCGTCATCGTCGAAATGGGC-3′
Slug	Forward	5’AAGCATTTCAACGCCTCCAAA3’
	Reverse	5’GGATCTCTGGTTGTGGTATGACA3’
Snail	Forward	5’TCGGAAGCCTAACTACAGCGA3’
	Reverse	5’AGATGAGCATTGGCAGCGAG3’
Twist	Forward	5’CCGGAGACCTAGATGTCATTG3’
	Reverse	5’CCACGCCCTGTTTCTTTG3’
